# The Psychophysiological Implications of Soundscape: A Systematic Review of Empirical Literature and a Research Agenda

**DOI:** 10.3390/ijerph16193533

**Published:** 2019-09-21

**Authors:** Mercede Erfanian, Andrew J. Mitchell, Jian Kang, Francesco Aletta

**Affiliations:** UCL Institute for Environmental Design and Engineering, The Bartlett, University College London (UCL), Central House, 14 Upper Woburn Place, London WC1H 0NN, UK; andrew.mitchell.18@ucl.ac.uk (A.J.M.); f.aletta@ucl.ac.uk (F.A.)

**Keywords:** soundscape, physiology, acoustic environment, perceptual attributes, auditory, noise

## Abstract

The soundscape is defined by the International Standard Organization (ISO) 12913-1 as the human’s perception of the acoustic environment, in context, accompanying physiological and psychological responses. Previous research is synthesized with studies designed to investigate soundscape at the ‘unconscious’ level in an effort to more specifically conceptualize biomarkers of the soundscape. This review aims firstly, to investigate the consistency of methodologies applied for the investigation of physiological aspects of soundscape; secondly, to underline the feasibility of physiological markers as biomarkers of soundscape; and finally, to explore the association between the physiological responses and the well-founded psychological components of the soundscape which are continually advancing. For this review, Web of Science, PubMed, Scopus, and PsycINFO were searched for peer-reviewed articles published in English with combinations of the keywords ‘soundscape’, ‘environmental noise/sound’, ‘physiology/physiological’, ‘psychology/psychological’, and ‘perceptual attributes/affective/subjective assessment/appraisals’. Previous research suggests that Electrocardiography (ECG) and Vectorcardiography (VCG) biometrics quantifying Heart Rate (HR), stimulus-locked experimental design, and passive listening with homogeneous populations are predominantly applied to characterize the psychophysiology underlying the soundscape. Pleasantness and arousal are the most frequent psychological descriptors for soundscape subjective appraisals. Likewise, acoustic environments are reported to inconsistently evoke physiological responses with great variability among studies. The link between the perceptual attributes and physiological responses of soundscape vary within and among existing literature. While a few studies detected a link between physiological manifestations of soundscape and the perceptual attributes, the others failed to validate this link. Additionally, the majority of the study findings were limited to one or two physiological responses.

## 1. Introduction

Sound is capable of generating powerful reactions in human beings which inform how they interact with and interpret their everyday environments. There is accumulating research indicating how sound affects the activity and functionality of the Central Nervous System (CNS) and the Peripheral Nervous System (PNS) in response to various forms of sounds, such as music [1,2], natural sounds [3,4], and urban sounds (or anthropogenic) [4,5] throughout a person’s lifetime [6,7].

From a physiological point of view, a sound signal is a detectable change in our external environment that can elicit a series of unconscious responses, unbalancing our homeostasis (the dynamic state of equilibrium). These responses are similar among populations in a similar situation, are automatic, and regulated by the Sympathetic Nervous System (SNS). The Parasympathetic Nervous System (PSNS), on the contrary, is constantly active to maintain homeostasis. It has been long established that both SNS and PSNS are part of the Autonomic Nervous System (ANS), which is per se a division of the Peripheral Nervous System (PNS) [8]. At the neural level, there appear to be segregated pathways, carrying the auditory signals and brain regions that orchestrate the way the brain responds to the auditory stimuli. The ascending auditory system, from the cochlea to the auditory cortex, is proven to respond to physical or acoustic properties of sound regardless of the emotional content or context of the sound (arousal) (the descending auditory pathway follows a similar path but in reverse) [9]. However, regions in the limbic system (emotional brain), such as amygdala and insula (known as a core hub of the “salience network”), are involved in processing the emotional magnitude of the auditory stimuli (valence), as a part of the CNS response [10,11]. The integration of the acquired auditory information from both ascending pathways results in the perception of the sound signals.

The soundscape, conceived as the acoustic equivalent of the landscape, is defined as the human’s perception of the acoustic environment, in context [12,13,14,15]. The soundscape can be the result of a single sound or a combination of sounds that arises from an engaging environment. The Canadian composer and naturalist R. Murray Schafer led much of the original work to advance research in the area [15], borrowing the term originally from work carried out by city planner Michael Southworth [16]. Since Schafer, there have been several multi-dimensional classifications for soundscapes. However, according to Schafer, the main components of the soundscape consist of keynote sounds, sound signals, and soundmarks [15]. The soundscape ecologist Bernie Krause characterized soundscapes into three main domains based on the source of the sound. According to his classification, the soundscape refers to a wide spectrum of sounds, encompassing natural sounds relating to non-organic elements of nature such as waterfalls (geophony), organic but non-human sources such as animals’ copulatory vocalizations (known as biophony), and all environmental sounds generated by human sources (anthrophony) such as human voices or human activity-related sounds [17,18,19].

Humans and soundscapes have a dynamic bidirectional relationship—while humans and their behaviour directly influence their soundscape, humans and their behaviour are in turn influenced by their soundscape. Several scientific communities in the area of neuroscience and psychology, therefore, have begun to pay close attention to our day-to-day exposure to particular sounds and their impact on the mental and physical health of individuals. Researchers in the areas of acoustics, environmental psychology, and auditory neuroscience outline the adverse impact of noise or negative sounds on well-being in an attempt to improve modern living standards [20,21,22,23,24]. In this regard, evidence indicates that positively perceived sounds (e.g., natural sounds) are tied with a high quality of life and enhanced psychological and physical health [5,25,26,27]. Subsequently, Attention Restoration Theory (ART) argues the impact of nature (e.g., being exposed to natural sounds such as waterfalls) on humans improved cognitive performance and stress recovery [28,29,30,31]. Not only has spending time in nature been demonstrated to have positive effects on humans’ nervous system but it has also been shown that humans innately tend to seek connections with nature, a hypothesis known as Biophilia [32].

Within many acoustics-related fields, noise is defined specifically as *unwanted sound* [33,34]. However, the term has been used inconsistently and interchangeably across the studies reviewed here. Additionally, we refer to ‘soundscape’ as the perception of the acoustic environment in context. In order to maintain consistency within the field and for ease of understanding within this review noise and soundscape will be used only to refer to the definitions above.

Despite the accumulating evidence of the effects of sound on the human nervous system, the candidate peripheral and central mechanisms underlying its elicitation are far from understood. Although there is a line of robust literature in the areas of urban planning, architecture, noise control and health (linking noise to adverse mental and physical health which necessitates noise mitigation) pertaining to the assessment and design of soundscapes, the soundscape concept has only recently been introduced to the areas of psychophysiology and neuroscience.

As the development of the newly emerging body of psychophysiology and neuroscience research is a necessity, it does not trivialize the psychological approach that has considerably served to enhance our knowledge in the soundscape research domain.

The physiological and psychological approaches are two sides of the same coin in the realm of soundscape research, strongly interconnected and equally important. The psychological approach, in soundscape research, strives to depict the acoustic environment through the human behavioural pattern by borrowing a more deductive approach. It translates the underlying mechanisms into explicit behavioural manifestations, resulting from the perception of the acoustic environment.

The physiological approach investigates the impact of the acoustic environment through the investigation of fundamental mechanisms of CNS and PNS by adopting a more inductive inferential approach. The physiological approach delineates the causation of the particular behaviour evoked by the environmental sounds.

The soundscape is composed of three main components—human interaction, acoustic environment, and perception—so it potentially draws attention across several life-science disciplines such as environmental psychology and public health, psychophysiology and auditory neuroscience. To the best of authors’ knowledge, no previous work has highlighted the explicit psychophysiological underpinnings of the soundscape. The current review of literature is the first work that reflects the fundamental mechanisms of the soundscape rather than its behavioural expressions.

Hence, the main research questions are (a) whether consistent methodologies were applied for the investigation of physiological aspects of soundscape; (b) secondly, what were the physiological markers as biomarkers of soundscape; and (c) finally, whether there was an association between the physiological responses and the perceptual attributes evoked by the acoustic environment.

## 2. Materials and Methods

The present literature review followed and was reported in accordance with PRISMA, Preferred Reporting Items for Systematic Review and Meta-Analysis (PRISMA) [35].

The inclusion criteria were: (a) including at least one measure of psychological dimensions or perceptual attributes of the soundscape as defined in ISO 12913-2; (b) including at least one physiological measure; and (c) published in English, excluding conference proceedings and books. The rationale of including these journal articles was that they all investigate the SNS and PSNS responses evoked by the acoustic environment.

The databases Thomson Reuters Web of Science, MEDLINE/PubMed, Scopus, and PsycINFO were searched for electronic records and studies related to the physiology of soundscape or the perception of environmental sounds, using the following Boolean search terms: ‘soundscape’ OR ‘environmental noise/sound’ AND ‘physiology/physiological’ AND ‘psychology/psychological’ OR ‘perceptual attributes’ OR ‘affective/subjective assessment/appraisals’ mentioned in either the title or the abstract. The time limitation was considered from 1 January 1990 to 1 February 2019, since the existing research is scarce. Journal articles were identified if they collected physiological responses to acoustic environments, regardless of whether they were investigating positive or negative soundscapes. One of the main theoretical frameworks on perceptual attributes has been proposed by Axelsson and his colleagues. Their circumplex model entails two horizontal and vertical axes, representing two different components of the soundscape. The vertical axis demonstrates the eventfulness component, which implies the intensity of soundscapes (i.e., eventful vs. uneventful). The horizontal axis refers to the emotional magnitude of soundscapes, representing the annoyance or pleasantness components (i.e., annoying vs. pleasant) [36]. The perceptual attributes proposed in this model have recently been adopted as a recommended standard in ISO 12913-2. In this review of the literature, the authors elucidate the perceptual attributes of soundscapes according to the circumplex model, where arousal is line with eventfulness on the vertical axis and valence is in line with pleasantness on the horizontal axis.

**Figure 1:**
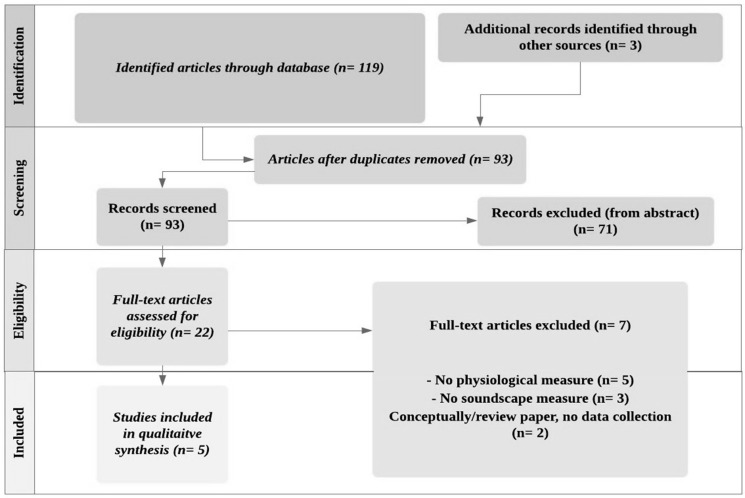
The flow of information through the different phases of the systematic review. The number of studies included in the qualitative synthesis (*n* = 5).

The search results were exported to Mendeley, which identified 122 published research articles. Then, the duplicates were removed (*n* = 29). Next, if the title or abstract did not provide sufficient information or did not meet the selection criteria, they were removed (*n* = 71). Then, the remaining 22 full-text articles were assessed for eligibility—of which, 17 were excluded from further inclusion as they were deemed irrelevant (e.g., physiology of noise). Finally, the exclusion resulted in five relevant journal papers which were read and analysed manually for method and content—of which, the oldest article was published in 2004 [5,10,37,38,39]. Figure 1 shows the process of inclusion of reviewed papers.

In the first step of the screening process, the first author (ME) reviewed the titles and abstracts of the journal articles and manually excluded the articles that did not meet the above-mentioned criteria. The second step was selecting the studies in which (ME) assessed the full-text articles for eligibility in accordance with the method outlined in PRISMA. Disagreements between the authors regarding the study selection were resolved by consensus.

## 3. Methodology in the Reviewed Studies

Although there is a long line of multidisciplinary research on soundscape, the majority of the available literature on soundscape and well-being has focused on the perceptual attributes and psychological components, lacking the conceptualization of its underlying physiological and neural mechanisms’ validity [5,40,41]. In other words, the current understanding of the entanglement of psychology and soundscape is mostly limited to the relationship between acoustic characteristics and the subjective appraisal of soundscapes, without the clarification of corresponding responses in the CNS or PNS. Studies focusing on identification of the physiological and neural mechanisms underlying soundscape are scarce and this mode of study warrants deeper and more elaborate investigation. In this section, we review the aims and methodologies of the included studies. The methodology includes the type of individuals included as the study sample, the experimental design, the auditory stimuli, and the subjective and objective measurements. Table 1 demonstrates the summary of the articles included in the review with detailed information on methods and the materials.

### 3.1. Study 1

Alvarsson et al. [5] were the first to report that the SNS gains a faster recovery while exposed to nature sounds compared to other sounds. This study aimed at comparing the effects of different sounds on the physiological recovery of individuals with induced psychological stress.

In this study, Alvarsson et al. tested university students in a three-part study. The first part began with one 5 min silence baseline, then five 2 min sections of testing or stressor, each followed by a 4 min period of relaxation or recovery. The authors made a 4 × 4 mixed design with relaxation sound as the within-subject variable and the order of the four different sounds as the between-subject variable. A Latin square matrix was applied in which the participants were assigned at random. The participants were given a mental arithmetic task as a stressor. In the task, the participants should decide whether the equation which appeared on the screen was true or false and received feedback as to whether their answer was ‘false’, ‘correct’, or ‘too late’.

The participants were exposed to 4 min samples of nature sounds (mixture of fountain and bird sounds, 50 dB L_Aeq, 4 min_), high noise (traffic noise, 80 dB L_Aeq, 4 min_), low noise (the same traffic noise adjusted to 50 dB L_Aeq, 4 min_), and ambient sound (referred to in the text as ‘ambient noise’) such as backyard sound including ventilation noise, 40 dB L_Aeq, 4 min_) during the recovery periods.

The indication of SNS activation was measured by Skin Conductance Level (SCL) and the recovery period or PSNS activity was measured by High-Frequency Heart Rate Variability (HF HRV). The perceptual attributes assessment was measured by a scale on three dimensions of pleasantness, eventfulness, and familiarity.

We should note that the exponential function coefficients in this work as part of the regression analysis produce a curve which is a factor of 10 off from the data. It is assumed this is a typographical error in the figure (where, e.g., b_3_ = −0.1111 should be b_3_ = −0.01111) that does not substantially impact the conclusions of the analysis.

### 3.2. Study 2

Gomez and Danuser [37] investigated the link between physiological parameters with subjective reports evoked by the acoustic environment. The main aim of this study was to evaluate the link between the judgment of affective arousal and valence and physiological responses.

The participants were selected mostly among university students. The authors exposed subjects to 30 s of mixed auditory stimuli that varied in emotional valence and arousal, representing environmental acoustic stimuli (referred to in the text as ‘environmental noises’) such as people playing tennis, sirens, as well as Western instrumental music (ranging from quiet Classical music, ‘Adagio assai’, M. Ravel, to loud Metal, ‘Black Arrows’, Manowar) with little acoustic variation over their 30 s, in order to maintain high intra-stimulus homogeneity.

All the environmental sounds and music excerpts were ranked from low to high based on a combination of the mean valence and mean arousal level, into groups (1–4, 5–8, 9–12, 13–16). Importantly, to ensure ecological validity of the auditory stimuli (ecological validity refers to methods and materials and the setting of the study that is close to the ‘real world’), the authors did not change or modulate the intensity of the soundscapes presented in the experiments.

The physiological responses were extrapolated from SCL, Respiration Rate (RR), and ECG. The affective rating was probed by a 9-point self-assessment Manikin on two dimensions of arousal and valence.

### 3.3. Study 3

Irwin et al. [10] explored the physiological validity of perceptual aspects of the acoustic environment. The purpose of the study was to assess the visceral and neural basis of cognitive and emotional responses to positive or ‘naturalistic urban soundscapes’ and their association with the perceptual dimensions (here, ‘naturalistic urban soundscapes’ refers to realistically recorded sound environments, not to urban soundscapes with a high proportion of natural sounds).

In this study, only native English speakers were selected. Here, we will only report the results of the physiological investigation as the outcome of neural responses is not in the scope of this review. For the physiological experiments, the authors selected a set of 8 s stimuli that would broadly range over the pleasantness and vibrancy scales.

A set of 219 recordings were selected from different archives and a selected set of 150 sounds was presented to the subjects in a randomized fashion and -in four sequential blocks lasting about 10 min each. Each block comprised a set of sounds with a duration of 8 s followed by 8 s or 16 s of silence. This study did not include any active task and subjects were instructed merely to remain still and listen to the samples. The visceral changes were registered by measuring HR. The perceptual attributes were tested by a self-report assessment on two dimensions of vibrancy and pleasantness.

### 3.4. Study 4

A more recent study by Hume and Ahtamad [38] employed a similar stimulus selection and presentation technique towards developing a better understanding of the physiological manifestation of soundscape and its possible relationship with subjective reporting of pleasantness and arousal. This study entailed three main objectives: to investigate (a) whether there is a change in registered physiological responses to different soundscapes; (b) whether there is a link between the pattern of physiological changes and the subjective rating of the pleasantness and arousal of the sound excerpt; and (c) whether there is a gender difference in any physiological responses.

They selected their participants among unpaid volunteers. The authors state that a key experimental design consideration was to limit the total time needed for each subject’s experiment to 20 min in order to mitigate potential issues with the experiment and listening fatigue. They also state that the presentation of 8 s sound stimuli was carefully considered in order to limit the prompting of the startle reflex in response to sudden loud sounds. The stimuli consisted of a range of sound-clips with a wide variety or source types such as ‘horses hooves on the road’ and ‘jackhammer plus traffic noise’. The physiological alteration was measured by recording HR, RR, and Electromyography (EMG). The pleasantness and arousal were evaluated by a 9-scale self-report.

### 3.5. Study 5

Another study by Medvedev et al. [39] investigated the restorative potential of acoustic environment perception. The first objective was to examine the variations of physiological responses to a number of auditory stimuli after a stressful task.

In their first study, they included unpaid postgraduate students or members of staff. Initially, Medvedev et al. exposed participants to a thirty-minute sequence with 4 min stimuli. Each sequence entailed five stress periods of 2 min, followed by a stress recovery period of four minutes. During each stress recovery period, the participants were randomly exposed to one of the four different environmental sounds or a silence condition. The stimuli consisted of the sounds of a forest, sea wave, busy motorway, and construction site.

In their second study, the objective was to measure the same physiological responses of soundscape during the rest state. Only university students were involved in the second phase of the study. The authors, therefore, exposed their subjects to different 2 min sounds to determine the effects on the ANS by exposing the subjects to unpleasant sounds during the rest period.

The sound samples selected for the second part of the study were three unnatural, two natural, and one orchestra piece.

The participants’ SCL and HR, representing the ‘fight, flight, or freeze’ response, were measured in both phases of the study, followed by participants’ subjective ratings of their perception of the four presented sound samples. The perceptual attributes in this study were arousal, dominance, eventfulness, familiarity, and pleasantness which were measured by a self-rating questionnaire.

### 3.6. Overall Methodological Approaches

The research on the physiological underpinnings of the soundscape is limited and so is the methodology. With respect to the available literature, the majority of the reviewed studies focused on the impact of the acoustic environment on ANS arousal by looking at specific physiological indicators within homogeneous populations, e.g., university students. The most commonly administered study design was passive listening with event-related responses (also known as stimulus-locked design) in which the participants’ physiological and perceptual responses, evoked by environmental sounds, were monitored. In the event-related design, the physiological arousal in response to environmental sounds is measured and evaluated in comparison with their baseline (silence condition). This response is generally more accurately related to the event in higher-frequency measures such as Electroencephalography (EEG), and less so in slower measures such as HR and Electrodermal Activity (EDA). Human, natural, and mechanical sounds reported being commonly used in most of the studies. HR is predominately applied for the quantification of ANS reactivity to environmental sounds. Analysis of heart rate (variability) is acknowledged as a cost-effective method with high validity and reliability, independent of risk factors of any cardiovascular diseases [42]. It has been commonly used in several studies to investigate the ANS reactivity to sounds [10,11,37]. The perceptual attributes of pleasantness (valence) vs. eventfulness (arousal) were mostly used among the existing literature.

## 4. Physiological Manifestations of Soundscapes

Beyond studies with a primary aim of investigating the psychological markers of the soundscape, a small body of research offers important insights toward a better understanding of the biomarkers of soundscapes by quantifying the physiological manifestations induced by ANS reactivity such as HR/HF HRV, EDA, RR, and musculoskeletal responses.

### 4.1. Heart Rate (HR) and Heart Rate Variability (HRV)

HR is a primary indication of ANS activation which is regulated by SNS and PNS. HR (V) are widely used in medical examinations and can be evoked in response to a variety of factors such as exercise, emotional arousal, and stimulant exposure. Although the outcome of HR and HRV are closely interconnected, they differ from each other in terms of pre-processing and analysis. While HR is measured in beats per minutes, or the average of the beats in a specific the time period (e.g., 1 min), HRV measures the specific change/variability in the time between the beats [43,44]. Only Alvarsson et al. applied HRV in the study. Notably, Bradley and Lang, in their 2000 study, pointed out a consistent ‘triphasic’ heart rate waveform across valence categories, in which an initial deceleration was followed by acceleration and then a secondary deceleration. When limited only to high-arousal sounds, the characteristics of the triphasic response varied across the valence categories [43].

In the 2004 study by Gomez and Danuser [37], the outcome of a mixed regression analysis of ECG indicated that high-arousal sounds (e.g., siren) evoked higher HR than low-arousal sounds (e.g., people playing tennis) (ß = 1.18, *SE* = 0.48, *p* = 0.05), although the association was not found between music and HR [37]. On the other hand, Medvedev et al., 2015 [39] in the second phase of their study found no relationship between HR and environmental sounds regardless of the sound source type (music, mechanical sounds, or natural sounds) and the findings of Irwin et al. [10] were shown to be limited to the immediate rise of HR right after the onset of the stimulus. However, the HR was then shown to have declined by a sustained reduction after a few seconds. Surprisingly, the outcome from Hume and Ahtamad’s study [38] is contrary to the previous works, showing a lowered HR in response to the sound stimuli, with a more prominent response to unpleasant sounds.

Although there are inconsistent findings with respect to HR response to soundscapes given a passive listening condition, there is a consensus between the two available studies which employed a stress task condition. Neither Medvedev et al.’s first study nor Alvarsson et al. could validate a significant link between HR and sound exposure during the recovery period after presenting a stress task [5,39].

### 4.2. Respiration Rate (RR)

Respiration rate (RR) is a vital sign and a widely used metric in medical research as a measurement of SNS reactivity and physiological stability of individuals with high accuracy. Abnormal RR is a serious indication of an imminent health crisis [45].

The study by Hume and Ahtamad [38] demonstrated an increased RR, to differing degrees, in response to all soundscapes except ‘man hiccupping’. Further investigation pointed to a gender difference in RR response in which men exhibited a greater increase of RR to all soundscapes compared to women [38]. These findings were in line with the previous study by Gomez and Danuser in which they discovered increased RR in both music and environmental sounds conditions [37]. A conclusion can be drawn when considering the small number of reviewed studies using the RR measure. These two studies mainly showed a consistent RR outcome, and they do highlight the respiratory candidate process for further research in peripheral nervous system activity associated with exposure to environmental sounds.

### 4.3. Electrodermal Activity (EDA)

EDA is a reliable and valid psychophysiological expression of SNS arousal. Physiologically, the activation of the endocrinology system is driven by the interconnection between CNS and PNS, especially by the sympathetic branch of ANS. The increased reactivity of SNS in the epidermis changes its electrical conductance, forming the basis of the EDA that is measured by Skin Conductance Response (SCR) (also known as Galvanic Skin Response (GSR)) and/or Skin Conductance Level (SCL) [46]. However, SCR reflects the rapid phasic component of EDA and SCL is derived from the slow and background tonic component.

The second study by Medvedev et al. showed no significant effect of sounds on SCL [39] which is almost in agreement with the study by Gomez and Danuser. In the latter study, the results of SCL showed that only ‘high’ music pieces (e.g., heavy metal) cued higher SCL compared to ‘low’ music (e.g., classical music), while SCL showed no significant relationship with environmental sounds [37].

The results of SCL in stress task studies are in less agreement than those with no stress task. Medvedev et al. failed to show significance in measured SCL changes in response to sounds after stress tasks, similar to their findings with no stress task. On the other hand, Alvarsson et al. demonstrated that subjects exposed to natural sounds recovered significantly faster (9–36% faster) from psychological stress in comparison to when they were exposed to ‘high noise’ (traffic noise, 80 dB L_Aeq, 4 min_). The authors reported that during exposure to high noise, a minor upturn in SCL was observed during the last 50 s of the 4 min recovery period, implying long exposure to the unpleasant sounds increased arousal [5]. All in all, studies into EDA response have been more consistent than other indications of ANS arousal in response to sound. However, the implications of EDA in the context of public health and well-being is still not clear.

### 4.4. Musculoskeletal Activity

The SNS modulates the functions in the musculoskeletal system (e.g., metabolism and locomotion). EMG measures musculoskeletal activity, from which SNS activity can be derived. The research on musculoskeletal response to soundscape has been confined to Hume and Ahtamad’s work. They showed that there was a significant relationship between attenuated muscle tone and pleasant sounds (e.g., evening bird songs with some traffic sounds). However, the significant difference was not consistent among all soundscape excerpts, and no difference between male and female participants could be detected [38].

## 5. The Association between Perceptual Attributes and Physiological Responses

The ANS is sensitive and responsive to external stimuli such as olfactory, textile, gustatory, vision, and auditory. The reactivity of ANS, triggered by environmental sensory stimulation, manifests in the variation of several systems in vivo, for instance, cardiovascular systems [47]. However, the representation of ANS reactivity does not always identify the type of emotion or perception, arising from this reactivation. It is therefore fundamental to make sense of the alteration of ANS through the subjective assessments which can be inconsistent. Table 2 shows the main objectives of the reviewed studies and their key findings.

### 5.1. Study 1

The outcome of the perceptual evaluation of the sounds in the study by Alvarsson et al. indicated that natural sounds were perceived as more pleasant than human and mechanical sounds. Among the latter, low traffic noise and the ambient sound were reported to be perceived as equally pleasant whereas the high traffic noise was reported to be the least pleasant sound. Further analysis showed that the high traffic noise was reported to be the most eventful while the ambient sound was rated as the least eventful and least familiar among all. The rationale of the authors for this finding was that the ambient noise contained no distinctive sound sources and may not be sufficiently salient to be differentiated [5]. This suggests that sympathetic arousal is influenced by the type of sound present in the environment with the largest observed differences between natural sounds and high traffic noise [5].

### 5.2. Study 2

The results of emotional magnitude ratings of both environmental sounds and music in the study by Gomez and Danuser showed wide variability in subjective ratings of valence and arousal. For the environmental stimuli, the subjective appraisals tended toward negative valence and high arousal. On the contrary, for the music condition, the subjective appraisals tended toward positive valence ratings and high arousal. In both conditions, faster breathing and high minute ventilation (MV) were related to increased arousal. While this association was reported to be present within the whole emotional magnitude (valence) for music, it was limited to only positive stimuli for the environmental sounds. However, their findings showed that slower breathing, as well as declined MV, were attributed to low-arousal sounds.

The thoracic breathing responses to environmental sounds and musical excerpts were demonstrated to be regulated moderately in line with valence ratings and arousal. The authors did not find any link between other physiological responses and perceptual attributes [37].

### 5.3. Study 3

Irwin et al. could not find a significant HR change associated with pleasantness and vibrancy. The results stayed the same for the association between pleasantness and the length of the sounds [10].

### 5.4. Study 4

In Hume and Ahtamad’s study, the HR and RR outcomes were reported to be greater in response to increased pleasantness and showed a clear gender difference with larger RR and HR responses observed for males [38]. Furthermore, the results indicated that decreased muscle activity derived from EMG was associated with more pleasant sounds with no gender effect.

### 5.5. Study 5

The outcome of the self-reported subjective ratings of the sound stimuli in the study by Medvedev et al. showed substantial variability of the mean response in the perceptual dimension assessing the appropriateness of the selected sounds. Among the presented sounds, ocean and birdsong were reported to be the most pleasant and arousing while construction and traffic were rated as the most dominating and eventful. The results of SCL in the first phase of the study, showed a faster decline in electrodermal activity in response to the more pleasant and less eventful sounds, although the perception of eventfulness and pleasantness differed among the participants. Likewise, sounds associated with the most familiarity and least eventfulness were linked to a decline of SCL. In addition, there was a significant difference in mean HR for the recovery period limited to only the eventfulness domain [39]. In the second phase of their study, the augmentation of SCL was reported to be attributed to the least pleasant and familiar as well as the most dominant sounds. Eventfulness and arousal showed no significant relationship with SCL change. Lower HR was significantly related to the least pleasant and the most familiar sounds.

### 5.6. Overall Association between Perceptual Attributes and Physiological Responses

Altogether, physiological expressions of the soundscape are not always aligned with the perceptual attributes and may remain inconclusive. Notably, it appears auditory stimuli length makes a major contribution to physiological expression and their link with perceptual attributes.

## 6. Discussion

The perception of the acoustic environment is the result of ongoing, meticulous, and unconscious interactions between subcortical and cortical brain structures. Consequently, the perception of the acoustic environment is closely correlated with the physiological properties elicited by the acoustic environment. There is a pressing deficiency in the existing soundscape research in which the implication of physiological changes in the context of human health and well-being is missing.

### 6.1. Soundscape and Noise Studies

Physiological studies have constituted a vital underpinning of conventional approaches to environmental noise control. As the direction of noise control has become progressively more holistic and begun incorporating the soundscape approach, an equivalent understanding of the physiological manifestations of the soundscape is needed. Since research on the physiology of noise pollution is generally more advanced than within the realm of the soundscape, in order to elucidate the physiological underpinnings of environmental sounds the authors briefly look into the physiology of noise exposure and its implications in soundscape research.

#### 6.1.1. Heart Rate

The majority of the existing body of literature in the physiology of soundscape is limited to HR, which currently indicates that noise or negatively perceived sound exposure significantly contributes to causing tachycardia [10,37]. However, the results are not consistent throughout the studies [5,38,39]. Looking back at the long-established literature in the area of noise pollution and health, we find similar findings. A study by Hsu et al. on forty patients recovering from anaesthesia underlines a positive significant association between an increase in noise intensity and HR [48]. It is important to note that in this study, the sound level was the only considered physical factor irrespective of other acoustic characteristics of the considered sounds.

Another study by Elbaz et al. investigating the effect of aircraft noise on HR during sleep shows that increased HR is linked to an increase in Sound Pressure Level (SPL) [49]. These findings are also in agreement with more prolonged conditions such as individuals exposed to occupational noise such as those investigated in a study by Burns and her colleagues. In this study, the test results of 57 electronic waste recycling workers indicated a significant rise in HR associated with work-related noise [50]. Abdelraziq et al. conducted another study investigating the effects of noise pollution on HR of school children. A strong positive correlation was found between augmented HR and noise pollution level [51]. In all, it seems that the amplitude level plays a crucial role in the magnitude of an evoked increase in HR as long as the sound is considered/labeled as noise.

#### 6.1.2. Respiration Rate

RR is a physiological metric with mainly consistent results in response to positively and negatively perceived extrinsic auditory stimuli. Regardless of the intensity of the sound, soundscape studies point to an increase of RR evoked by the acoustic environment [37,38]. Seemingly, RR alteration is a fundamental physiological parameter, indicating the impact of noise on homeostasis. Hassanein et al. studied the impact of interrupted loud noise in the Neonatal Intensive Care Unit (NICU). In this study, they registered the neonatal physiological responses at a different time of day and during different sound events. The findings revealed a significant increment of RR in pre-term neonates in comparison with full-term neonates when exposed to sound events during the day [52].

The existing literature also looked into the long-term effects of noise (e.g., road traffic) on physical health in which an association between traffic noise exposure and respiratory morbidities [53] was demonstrated. According to the literature, the adverse effects of exposure to noise are a high risk of developing diseases such as bronchitis and asthma and pneumonia in children that was attributed to an increase of 1 dB (A) of daily noise levels, as a short-term association [54].

#### 6.1.3. Skin Conductance Response/Level

The essence of SCL response in soundscape research is to infer psychological descriptors from measured induced EDA. Whereas EDA has been proved to be an accurate manifestation of SNS activation, it does not always show a consistent outcome throughout the soundscape physiology research [5,35,37]. On the other hand, the majority of the findings on EDA when restricted to the response to noise is in agreement. A study by Park and Lee tested the effects of ‘floor impact noise’ on the physiological responses. Although the floor noise seems to be a trivial sound and not salient enough to be perceived as annoying, it leads to a significant increase in EDA of the subjects [55]. Similarly, Glass and Singer found a rise in the SCR evoked by noise in more than 90% of their participants which was irrespective of the amplitude and unpredictability of the sound. However, some further and deeper investigation revealed a drop in SCR which was an indication of the habituation in almost 90% of their participants, while only 4% seemed to be unable to adapt physiologically [56].

#### 6.1.4. Musculoskeletal Responses

The potential for the examination of soundscape via musculoskeletal system activity is far from limited and understanding it should be deemed highly necessary. EMG, an electrodiagnostic technique which can help to better understand the possible effect of sounds in the musculoskeletal system is limited to one study in the context of soundscape but has been shown to increase in response to pleasant soundscapes [38]. The large body of literature on skeletal muscle activity provoked by noise is in line with the responses found by Hume and Ahtamad. A study by Trapanotto and colleagues in the Paediatric Intensive Care Unit (PICU) revealed an increase in skeletal muscle tone detected by EMG, in particular during exposure to highly intense noise [57].

In short, the literature on soundscape and noise appear to have consensus regarding the increase in HR, with less consistency in results among soundscape studies. The RR and EMG outcomes showed more steady patterns in both areas of research indicating the increment of RR in response to sound stimuli, with consideration of the limitation of RR application among soundscape research. On the other hand, there is less agreement on the results of EDA between soundscape and noise research domains since the findings are uncertain among soundscape literature.

### 6.2. Suggestions for the Research Agenda

#### 6.2.1. Soundscape Characterization

The limited studies on physiological indices predominantly focus either on the adverse or the favourable aspects of soundscapes. Consequently, an interdisciplinary and holistic probe is deemed necessary to shed light on the entire spectrum spanning positive and negative soundscapes in order to better understand the impact of soundscapes in a general context. Since there is no sufficient evidence to make conclusions about either physiological manifestation related to soundscapes, we suggest that future investigations classify/categorize the types of sound sources. These categories (e.g., human, nature, and mechanical) would function as a steady baseline to which we can attribute the negative and positive responses [58,59]. Investigating soundscapes based on the source of the sound and not how positive or negative they are perceived will facilitate the researchers to characterize the physiological and neural responses regardless of the variability in the listeners [60].

Considering distinctions in the physiological responses to single or complex sound sources would also enable future research to better grasp the differences in potential evoked biological responses when considering the different source type characterizations suggested above.

Another essential aspect of soundscapes that may lead to invaluable findings and which could strongly reflect on human physiology is the duration of exposure and temporal variation of the acoustic environment, in an experimental context, reflecting in physiological responses [61,62]. The outcome could be potentially translated to how ANS habituates or sensitizes to sounds in the real world. Future research should take these factors into consideration because the current body of research strongly implies that sound duration and variation, in general, are well-established contributors to nervous system adaptation and the resulting habituation or sensitization. Although the habituation or sensitization of the nervous system to sounds relies upon acoustic features of the presented sound [63], to date they have not been addressed in the context of soundscapes.

#### 6.2.2. Recording and Reproduction of Soundscapes

Given that many of the most promising physiological metrics must be performed in a laboratory setting (e.g., RR), the ecological validity of reproduced soundscapes is a vital consideration. Toward this, much work has been carried out within the field of soundscape studies toward reducing artificiality of the controlled acoustic environment and achieving immersion. Two main methods are used: binaural recordings and ambisonic recordings which use multiple microphones to record a sound field at a single point in full-sphere surround. Binaural audio has long been used in soundscape studies, but its use has generally been limited in physiological studies of soundscape and ambisonic audio has not yet been applied to physiological studies. Both binaural and ambisonic audio, which allows the full spherical sound field to be reproduced very close to what would be experienced in situ, can improve immersion and add ecological validity to laboratory studies [64].

In addition to the ecological validity of the acoustic environment, in order to fully research soundscape, it is highly recommended [65] to take the effects of other sensory modalities into account such as vision, olfactory, and environmental components such as temperature. The inclusion of other modalities represents another step toward understanding the cross-modalities interaction of perception of acoustic environment and how the interpretation of acoustic information is an integral part of this process by introducing new methods such as Virtual Reality (VR), Augmented Reality (AR) [66,67]. Toward this aim, virtual reality reproductions are increasingly being used to create an immersive and soundscape reproduction which can incorporate multiple sensory modalities [68].

#### 6.2.3. Physiological and Neural Models

The conceptual framework of the physiological and neural network may be fruitful in order to develop specific candidate physiological and neural mechanisms of soundscape. This approach emphasizes an understanding of basic brain processes at the network level. Applying a network level approach would allow testing hypotheses about different brain regions working in an integrated and coordinated fashion. While the majority of our knowledge of how the brain implements soundscape processing is based on the assumption of assigning a certain role to each region of the brain, there is an emerging realization that shows this approach is not pragmatic in understanding brain function. Instead, it is suggested that functions of the brain in response to soundscape should be understood at the network level. In order to precisely pinpoint the physiological and neural mechanisms behind soundscape, it will not be enough to determine which brain substrates are activated by different sounds. We must also understand how those brain areas work together at a network level.

#### 6.2.4. Physiological and Psychological Models/Body–Mind Integration Model

The integration of physiological and psychological approaches known as the ‘body–mind’ model can substantially strengthen our understanding of how and why we experience the soundscape the way that we do. In this model, the mind and body are not seen as separately functioning entities, but as one holistic bidirectional unit, driven by top–down and bottom–up factors. It is for this reason that they consolidate each other’s results as a more complete scientific method. While the psychological component of the soundscape model relies on a wide range of environmental variables such as socio-cultural factors [69,70,71], the physiological aspect holds more consistency among different populations [37,38,39]. Also, the methods of data collection for each approach are considerably different. The psychological studies of soundscape revolve around narrative interviews, soundwalks, and uncontrolled behavioural observations [69,72,73]. The physiological is limited to controlled lab experiments [5,10,37,38,39]. Put together, these two approaches are as complementary as they are mutually reflective and the necessity to develop both in parallel is self-evident.

#### 6.2.5. Relation to Health and Well-Being

Soundscape research could also benefit from ameliorating clarity and consistency in the description of the physiological and neurophysiological processes attributed to the soundscape in the context of human well-being. Notwithstanding a few existing studies which strove to unveil the physiological and neurophysiological indications of soundscapes [5,9,37,38,39], there is still very little known about what these research outcomes mean in terms of health and well-being. In other words, more research is needed to interpret the fundamental physiological alterations evoked by the acoustic environment in terms of acoustic comfort, health, and well-being to enable policy makers and professionals to extrapolate solid results. In addition, further research is needed toward ‘reverse translation’, in which characteristics of the acoustic environment can be extrapolated from the ‘unconscious mechanisms’ identified in physiological responses [74]. The success of this reverse translation approach could enable researchers to better predict physiological responses to measured sound environments and, finally, to predict overall health and well-being outcomes independent of subjective ratings.

## 7. Conclusions

The present literature review reported on the established psychophysiological outcomes of the soundscape within the existing research, such as HR, HRV, SCL, RR, and musculoskeletal activity underlying soundscape. Hence, a systematic review in accordance with the PRISMA guidelines was performed on four principle databases in the areas of psychophysiology and neuroscience. In this review, we raised three questions: what metrics were used to detect the physiological manifestations of soundscape; whether significant physiological changes occur in response to the acoustic environment; and ultimately, whether the detected changes correspond with perceptual attributes.

The outcome of the review indicates that HR measurement and an event-related or stimulus-locked design with passive listening are commonly applied among the existing soundscape physiology research, mostly with homogeneous subject populations. The most frequent subjective assessments used among the studies were based on two perceptual attributes of valence and arousal.

The reviewed literature showed the physiological manifestations of the soundscape with wide variability, ranging from rising to fall, which were inconsistently associated with the perceptual attributes. Based on the scope of the discussion provided in the papers themselves and the inconsistency of the results, conclusions cannot currently be drawn about a broader discussion regarding the implementation of the soundscape approach to the design of environments based on physiological manifestations or responses.

To advance the research on the psychophysiology of soundscape, we propose a potential classification for the environmental sounds with single and complex sources. It is worthwhile to look into the link of environmental sounds’ temporal variation and length and how they may affect the adaptation, habituation or sensitization of ANS. It is recommended to investigate the psychophysiology aspect of the soundscape in a network level, taking into account all the contributing CNS and PNS factors and how they interact with each other, particularly in the context of health and wellbeing.

While the study of physiology and neurophysiology of soundscape is still in its infancy and inconclusive, cross-disciplinary team collaboration is highly recommended to propose an optimum biomarker multi-model for the soundscape investigation. As acoustic design moves further toward the soundscape approach, so should the psychophysiology of soundscape advance, to provide the same underpinning as the physiology of noise provided for conventional noise control.

## Figures and Tables

**Table 1:** Summary of the review studies (*n* = 5).

Reviewed Articles	Stimuli Length	Number of Stimuli	Metrics	Sound Source Category				Physiological Metrics	Perceptual Attributes		
				Human	Natural	Mechanical	Music		Valence	Arousal	Other
**Gomez and Danuser, 2004**	30 s	32	52.2–77.5 dB (A) **	e.g., people playing tennis	-	e.g., siren	Ranging from low to high, e.g., Black arrows by Manowar	HR, SCL, RR	Valence	Arousal	-
**Alvarsson et al., 2010 ^†^**	4 min	N/A	40–80 dB (A)	-	from fountain and tweeting birds	road traffic (high, low and ambient)	-	HF HRV, SCL	Pleasantness	Eventfulness	Familiarity
**Irwin et al., 2011**	8 s	150	71 dB (A) *^,^**	e.g., giggling	e.g., wind	e.g., construction	-	HR, fMRI, PET	Pleasantness	Vibrancy	-
Hume and Ahtamad, 2013 ***	8 s	18	60–74 dB (A) **	e.g., vomiting	e.g., horse hooves on road	e.g., traffic noise	-	HR, RR, EMG	Pleasantness	Arousal	-
**Medvedev et al., 2015**	4 min	4	64 dB (A) *^,^**	-	e.g., ocean	e.g., road noise	-	HR, SCL	Pleasantness	Arousal, eventfulness	Dominance, familiarity
**Medvedev et al., 2015**	2 min	6	64 dB (A) *^,^**	-	e.g., ocean	e.g., road noise	-	HR, SCL	Pleasantness	Arousal, eventfulness	Dominance, familiarity

* Normalized Auditory Stimuli. ** L_eq_ Sound Pressure Level (SPL). *** Used sound-clips with mix sources. ^†^ Binaural Recording. HR, Heart Rate; HF HRV, High Frequency Heart Rate Variability; fMRI, Functional Magnetic Resonance Imaging; PET, Positron Emission Tomography; SCL, Skin Conductance Level; RR, Respiration Rate; EMG, Electromyography.

**Table 2:** Studies from 1990 to 2019 of the outcome of the psychophysiology of soundscape.

Reviewed Articles	Objective (s)	Research Design	No of Participants	Key Findings
**Gomez and Danuser, 2004**	(A) Evaluation of the link between the judgment of affective arousal and valence and physiological response	Stimulus locked	31	(A) Only RR in response to environmental sounds and music is in line with valence and arousal to a certain extent
Alvarsson et al., 2010 *	(A) Comparison of the effects of different sounds on the physiological recovery of individuals with induced psychological stress	Latin square matrix	40	(A) Nature sound accelerates the physiological recovery of SNS after psychological stress
**Irwin et al., 2011**	(A) Assessment of the visceral basis of cognitive and emotional responses to positive or ‘naturalistic urban soundscapes’ (B) Their association with the perceptual dimensions	Stimulus locked	16	(A) Increment of HR in response to the onset of auditory stimuli (B) No association was found
**Hume and Ahtamad, 2013**	(A) Investigation of variation in registered physiological responses to different soundscapes (B) Investigation of the link between the pattern of physiological changes and the subjective rating the pleasantness and arousal of the sound excerpt (C) Investigation of a gender difference in any physiological responses	Stimulus locked	80	(A) Decrement of HR, increment of RR, decrement of EMG limited to pleasant sounds (B) Increment of HR, and RR but decrement of EMG to rise of pleasantness, no association between physiological responses and valence reported (C) A significant rise in HR and RR responses in men but not in EMG
Medvedev et al., 2015 *	(A) Examining the variations of physiological responses to a number of auditory stimuli after a stressful task	Latin square matrix	45	(A) Faster recovery of SCL in response to the most pleasant and the least eventful sounds and a significant difference in mean HR only during the eventful sound
**Medvedev et al., 2015**	(A) Measuring the same physiological responses of soundscape during rest state	Stimulus locked	30	(A) SCL linked to the least pleasant, familiar, and dominating sounds specially in the first 10 s; the increase of SCL associated with the least pleasant, familiar and the most dominant; no SCL change in response to the most and the least arousing and eventful sounds; a fall in HR associated with the least pleasant and the most familiar sounds

* Stress Task.

## Abbreviations

The following abbreviations are used in this manuscript:

AR	Augmented Reality
CNS	Central Nervous System
PNS	Peripheral Nervous System
SNS	Sympathetic Nervous System
PSNS	Parasympathetic Nervous System
HR	Heart Rate
HRV	Heart Rate Variability
HF HRV	High-Frequency Heart Rate Variability
RR	Respiration Rate
EDA	Electrodermal Activation
EMG	Electromyography
PRISMA	Preferred Reporting Items for Systematic Review and Meta-Analysis
MV	Minute Ventilation
SCL	Skin Conductance Level
SCR	Skin Conductance Response
VR	Virtual Reality
